# Interventricular septal hydatid cyst protruding into both outflow tracts: images in cardiology

**DOI:** 10.1093/ehjcr/ytag558

**Published:** 2026-08-04

**Authors:** Vignesh R, Soumya Guha, Kumar Sharp, Sohini Majumdar

**Affiliations:** Department of Cardiology, Desun Hospital and Heart Institute, EM Bypass, Kolkata 107, India; Department of Cardiothoracic and Vascular Surgery, BM Birla Heart Research Centre, Alipore, Kolkata 27, India; Department of Cardiothoracic and Vascular Surgery, BM Birla Heart Research Centre, Alipore, Kolkata 27, India; Department of Cardiac Anesthesia, BM Birla Heart Research Centre, Alipore, Kolkata 27, India

## Summary

Cardiac echinococcosis is an uncommon disease, with an estimated prevalence ranging from 0.5% to 2%.^1^ As contractions of the heart cause natural resistance for the presence of viable hydatid cyst, primary echinococcosis of heart is a rare event. The distribution of echinococcosis in the heart depends on the blood supply to that part of the heart. Due to the rich coronary blood supply, the left ventricular wall is the most common cardiac location (60%), followed by the right ventricle (10%), pericardium (7%) left atrium (6–8%), and right atrium (3–4%). The interventricular septum (IVS) is less frequently involved. It is reported in just 4% of cardiac cases.^1^ Review of the PubMed database identified only 45 cases of hydatid cyst in the IVS reported between 1964 and 2019.^2^ We present a unique case of cardiac hydatid cyst located in IVS protruding into both LV and RV outflow, which was assessed through multimodality imaging and subsequently successfully excised surgically.

## Case description

**Figure 1:**
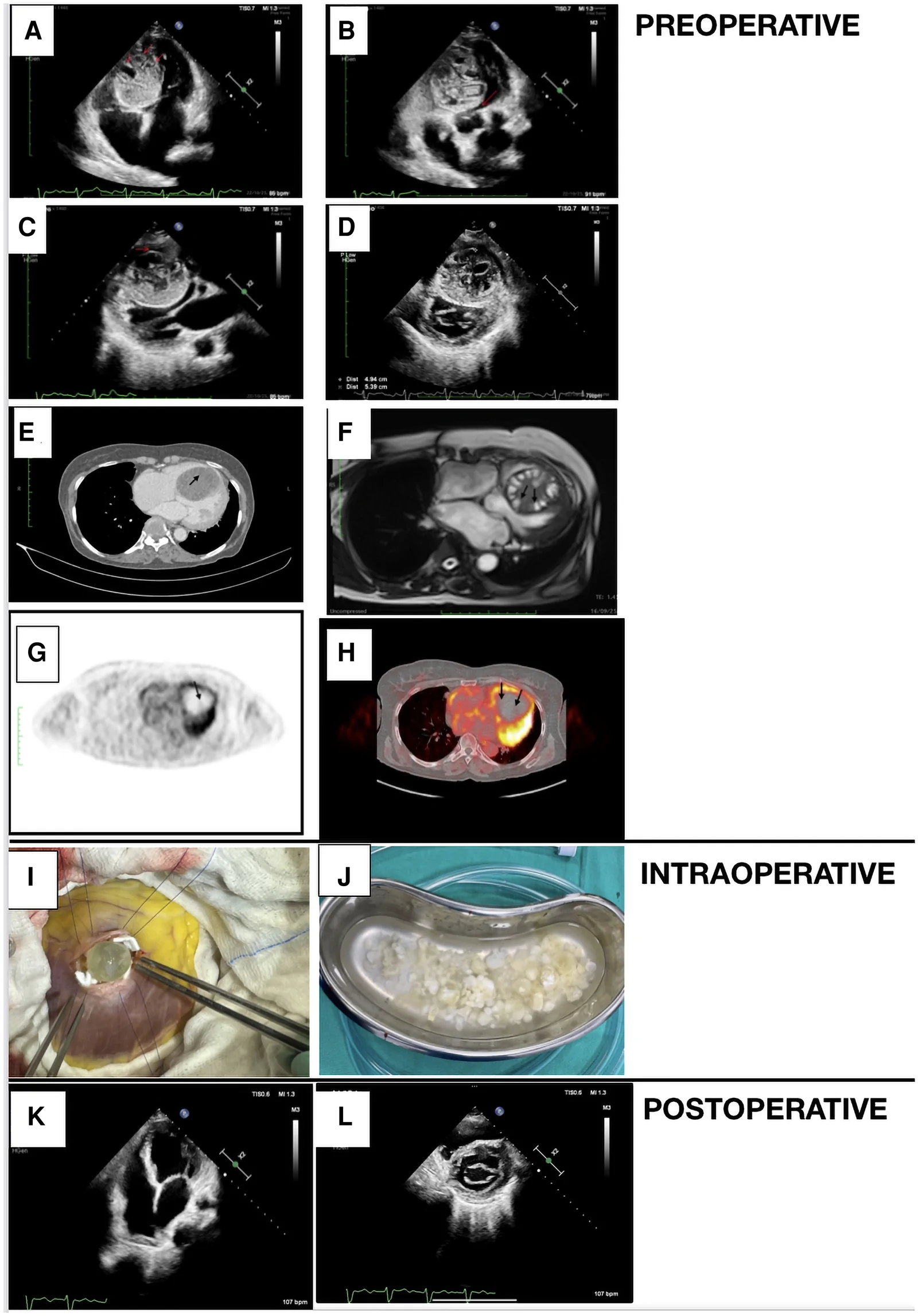
(*A* to *H*) preoperative multimodality imaging panel. (*A*) TTE A4C view showing cardiac mass in interventricular septum with arrows indicating multiple internal cysts with septations; (*B*) TTE A5C view showing mass protruding into LVOT (arrow); (*C*) TTE PLAX view showing mass protruding into proximal RVOT (arrow); (*D*) TTE PSAX view showing mass with multiple internal cysts and septations; (*E*) Computed tomography chest showing hypodense, nonenhancing mass in interventricular septum with regular margin without calcification with arrow indicating internal cysts with septations; (*F*) Cardiac magnetic resonance image showing sessile cardiac mass with no contrast enhancement in interventricular septum with arrows indicating multiple internal cysts with septations; (*G*, *H*) 18F-Fluorodeoxyglucose positron emission tomography-computed tomography (FDG PET-CT) scan showing cardiac mass with arrows indicating low-grade FDG avid soft tissue mass; (*I*, *J*) Intraoperative panel. (*I*) Lobulated, yellowish tan cardiac mass *in situ* handled during the resection; (J) Surgical specimen showing multiple cystic compartments with chalky white material; (*K*, *L*) Postoperative panel. (*K*, *L*) TTE A4C and PSAX view showing complete removal of hydatid cyst from interventricular septum. TTE: transthoracic echocardiography, A4C: apical four chamber, A5C: apical five chamber, PLAX: parasternal long axis, PSAX: parasternal short axis.

A 57-year-old female presented with dyspnoea and chest pain for 6 months and one episode of syncope. Physical examination, blood investigations, chest radiograph, and holter monitoring were unremarkable. Electrocardiogram revealed incomplete RBBB with T wave inversion from V2 to V6 (see Supplementary material online, Figure *S1*). Echocardiography disclosed interventricular septal cystic lesion with multiple internal septations bulging into both outflow tracts without doppler evidence of obstruction (*Figures 1A–D*; Videos *1*–*3*). Diagnostic echocardiographic mass (DEM) score was 4 points (1 point each for inhomogeneity, sessile, nonleft localization and invasiveness) suggesting malignancy (DEM score≥3 suggests malignancy).^3^ Cardiac CT revealed 66 × 46 mm non-enhancing, hypodense interventricular septal mass having regular margin, internal septations without calcification (*Figure 1E*); red flag sign was 2 points (1 point each for invasiveness and diameter > 30 mm) which was negative for malignancy (summatory of red flag signs≥5 suggests malignancy).^3^ CT lungs, abdomen, and brain revealed no masses. Cardiac MRI revealed sessile interventricular septal mass with no gadolinium enhancement, with internal septations (*Figure 1F*); CMR score was 1 (1 point for sessile mass), which was not suggestive of malignancy (CMR score≥5 suggests malignancy).^3^ 18F-FDG Whole Body PET CT revealed low-grade FDG avid soft tissue mass (*Figure 1G, H*) with SUV max of 6.2 (SUV max≥4.9 suggestive of malignancy),^3^ suggesting malignancy but ruled out, other location malignancies. Echinococcus IgG serology (Elisa) was positive. CAG revealed normal coronaries without external compression. Considering the location, size, embolism/anaphylaxis/arrhythmia risk, outflow tract protrusion, it was decided for surgical excision. Daughter cysts were excised (*Figure1I, J*; Video *1*) and pathological specimens confirmed hydatid cyst. She was on albendazole preoperatively and postoperatively for 6 months and remained asymptomatic. This case underscores the importance of multimodality imaging for detailed cardiac mass assessment, for deciding treatment modality.

## Supplementary Material

ytag558_Supplementary_Data

## Data Availability

All data are incorporated into this article.
